# Web alert: Microbes in drug delivery

**DOI:** 10.1111/1751-7915.14251

**Published:** 2023-03-23

**Authors:** Lawrence P. Wackett*

**Affiliations:** ^1^ Department of Biochemistry, Molecular Biology & Biophysics, BioTechnology Institute University of Minnesota St. Paul Minnesota 55108 USA

Microbe‐based drug delivery.

https://www.ncbi.nlm.nih.gov/pmc/articles/PMC6895346/

This review targets microbe‐based drug delivery with the goal of providing site‐specific medical benefits to patients.

Synthetic microbes as drug delivery systems.

https://pubs.acs.org/doi/10.1021/sb500258b

This article examines potential developments in medicine for using microbes to diagnose disease, produce therapeutics in situ, and deliver medicines.

Microbial‐fabricated nano‐systems.

https://www.frontiersin.org/articles/10.3389/fchem.2021.617353/full

This is a broad treatment on the formulations of microbes with metals, polysaccharides, and other agents for making nano‐scale delivery agents.

Modes of therapeutic delivery.

https://www.cell.com/trends/microbiology/fulltext/S0966‐842X(22)00249‐9

This review discusses bacterial therapeutics and vaccines, with a focus on those moving into clinical trials.

Bacteria and anti‐cancer drugs.

https://www.sciencedirect.com/science/article/pii/S0168365920303849

This review article highlights the use of bacteria or bacterial derivatives as drug carriers for cancer therapy.

Bacterial drug delivery IP.

https://www.wipo.int/wipo_magazine/en/2014/04/article_0002.html

This interesting article in the World International Patent Organization (WIPO) magazine discusses a microbial drug patented for the treatment of gastrointestinal disorders.

Bacterial lysing systems in cancer therapy.

https://www.nature.com/articles/s41467‐021‐26367‐9

This article deals with the use of a *Salmonella*‐derived delivery system to induce lysis and release proteins specifically into tumour cells.

Bacteria and medical implants.

https://www.mdpi.com/2079‐4983/13/4/173

This deals with bacterially responsive drug delivery for the release of antibacterial compounds at the site of implants to mitigate against infections.

Anti‐microbial peptide delivery.

https://agris.fao.org/agris‐search/search.do?recordID=US202000140612

Nano‐scale drug delivery systems are being investigated for the delivery of bacterially produced toxins called bacteriocins.

